# A New Spontaneously Diabetic Non-obese Torii Rat Strain With Severe Ocular Complications

**DOI:** 10.1155/EDR.2000.89

**Published:** 2000

**Authors:** Masami Shinohara, Taku Masuyama, Toshiyuki Shoda, Tadakazu Takahashi, Yoshiaki Katsuda, Kajuro Komeda, Masatoshi Kuroki, Akihiro Kakehashi, Yasunori Kanazaw

**Affiliations:** 1 Research Laboratories Torii Pharmaceutical Co., Ltd., 1-2-1 Ohnodai, Midori-ku Chiba 267-0056 Japan; 2 Division of Laboratory Animal Science Animal Research Center Tokyo Medical University Shinjuku-ku Tokyo 160-0022 Japan; 3 Omiya Medical Center Jichi Medical School Amanuma-cho Omiya 330-0834 Japan; 4 Japan Tobacco Inc. Toxicology Research Laboratories Central Pharmaceutical Institute 23 Nakogi, Hatano Kanagawa 257-0024 Japan; 5 Japan Tobacco Inc. Central Pharmaceutical Research Institute 1-1 Murasaki-cho Takatuki Osaka 569-1125 Japan

## Abstract

A new spontaneously diabetic strain of the Sprague-Dawley rat was established in 1997 and named the SDT (Spontaneously Diabetic Torii) rat. In this research, we investigated the characteristics of the disease condition in the SDT rats. The time of onset of glucosuria was different between male and female SDT rats; glucosuria appeared at approximately 20 weeks of age in male rats and at approximately 45 weeks of age in female rats. A cumulative incidence of diabetes of 100% was noted by 40 weeks of age in male rats, while it was only 33.3% even by 65 weeks of age in female rats. The survival rate up to 65 weeks of age was 92.9% in male rats and 97.4% in female rats. Glucose intolerance was observed in male rats from 16 weeks of age. The clinical characteristics of the male SDT rats were (1) hyperglycemia and hypoinsulinemia (from 25 weeks of age); (2) long-term survival without insulin treatment; (3) hypertriglyceridemia (by 35 weeks of age); however, no obesity was noted in any of the male rats. The histopathological characteristics of the male rats with diabetes mellitus (DM) were (1) fibrosis of the pancreatic islets (by 25 weeks of age); (2) cataract (by 40 weeks of age); (3) tractional retinal detachment with fibrous proliferation (by 70 weeks of age) and (4) massive hemorrhaging in the anterior chamber (by 77 weeks of age). These clinical and histopathological characteristics of the disease in SDT rats resemble those of human Type 2 diabetes with insulin hyposecretion. In conclusion, SDT rat is considered to be a potentially useful model for studies of diabetic retinopathy encountered in humans.


*Int. Jnl. Experimental Diab. Res.*, Vol. 1, pp. 89–100
Reprints available directly from the publisher
Photocopying permitted by license only

© 2000 OPA (Overseas Publishers Association) N.V.
Published by license under
the Harwood Academic Publishers imprint,
part of The Gordon and Breach Publishing Group.
Printed in Malaysia.

### A New Spontaneously Diabetic Non-obese Torii Rat Strain with Severe Ocular Complications

MASAMI SHINOHARA[a,*], TAKU MASUYAMA[a,†], TOSHIYUKI SHODA[a,†], TADAKAZU TAKAHASHI[a,†], YOSHIAKI KATSUDA[a,‡], KAJURO KOMEDA[b], MASATOSHI KUROKI[c], AKIHIRO KAKEHASHI[c] and YASUNORI KANAZAWA[c]

[a]*Research Laboratories, Torii Pharmaceutical Co., Ltd., 1-2-1, Ohnodai, Midori-ku, Chiba 267-0056, Japan;*
[b]*Division of Laboratory Animal Science, Animal Research Center, Tokyo Medical University, Shinjuku-ku, Tokyo 160-0022, Japan;*
[c]*Omiya Medical Center, Jichi Medical School, Amanuma-cho, Omiya 330-0834, Japan*

*(Received 24 October 1999; In final form 30 December 1999)*

**A new spontaneously diabetic strain of the Sprague-Dawley rat was established in 1997 and named the SDT (Spontaneously Diabetic Torii) rat. In this research, we investigated the characteristics of the disease condition in the SDT rats. The time of onset of glucosuria was different between male and female SDT rats; glucosuria appeared at approximately 20 weeks of age in male rats and at approximately 45 weeks of age in female rats. A cumulative incidence of diabetes of 100% was noted by 40 weeks of age in male rats, while it was only 33.3% even by 65 weeks of age in female rats. The survival rate up to 65 weeks of age was 92.9% in male rats and 97.4% in female rats. Glucose intolerance was observed in male rats from 16 weeks of age. The clinical characteristics of the male SDT rats were (1) hyperglycemia and hypoinsulinemia (from 25 weeks of age); (2) long-term survival without insulin treatment; (3) hypertriglyceridemia (by 35 weeks of age); however, no obesity was noted in any of the male rats. The histopathological characteristics of the male rats with diabetes mellitus (DM) were (1) fibrosis of the pancreatic islets (by 25 weeks of age); (2) cataract (by 40 weeks of age); (3) tractional retinal detachment with fibrous proliferation (by 70 weeks of age) and (4) massive hemorrhaging in the anterior chamber (by 77 weeks of age). These clinical and histopathological characteristics of the disease in SDT rats resemble those of human Type 2 diabetes with insulin hyposecretion. In conclusion, SDT rat is considered to be a potentially useful model for studies of diabetic retinopathy encountered in humans.**

*Keywords:* Type 2 diabetes, non-obese, Spontaneously Diabetic Torii strain, pancreatic fibrosis, cataract, diabetic retinopathy

### INTRODUCTION

Diabetes mellitus (DM) has become a global health problem; the incidence of the disease is graually increasing in all regions of the world.[1–3] The number of people suffering

*Corresponding author. Japan Tobacco Inc., Toxicology Research Laboratories, Central Pharmaceutical Institute, 23 Nakogi, Hatano, Kanagawa 257-0024, Japan. Tel.: +81-463-81-1277, Fax: +81-463-82-3444, e-mail: masami.shinohara@ims.jti.co.jp
†Present address: Japan Tobacco Inc., Toxicology Research Laboratories, Central Pharmaceutical Research Institute, 23 Nakogi, Hatano, Kanagawa 257-0024, Japan.
‡Present address: Japan Tobacco Inc., Central Pharmaceutical Research Institute, 1-1 Murasaki-cho, Takatuki, Osaka 569-1125, Japan.

from DM is expected to rise to 2.21 billion by the year 2010.[4] Diabetes is not a disease of any single organ. To clarify the complicated nature of the disease, studies using diabetic animal models are essential. In particular, the development of spontaneously diabetic animal models is very important to clarify the pathogenesis of DM and the pattern of development of complications in the human disease course. So far, many spontaneously diabetic animal models have been reported, including Type 1 models such as Bio-Breeding (BB) rats,[5] Non-Obese-Diabetic (NOD) mice,[6] Long-Evans-Tokushima-Lean (LETL) rats[7] and Komeda-Diabetes-Prone (KDP) rats.[8] Type 2 models such as *ob/ob* mice,[9] KK mice,[10] *db/db* mice,[11] Goto-Kakizaki (GK) rats,[12] Wistar fatty rats [13] and Otsuka-Long-Evans-Tokushima-Fatty (OLETF) rats [14] are also well known. These models, in which the pattern of progression and symptoms closely mimic those of DM in humans, play a significant role in diabetes research, even though any single model may be inadequate for clarifying all the issues related to the disease. We established a new inbred strain, the Spontaneously Diabetic Torii (SDT) rats, which survive for a long time with hyperglycemia, even without insulin therapy and may be useful particularly for the research of retinopathy. They exhibit tractional retinal detachment with fibrous proliferation of retinopathy similar to that occurring in human diabetes. In the present report, we describe the pathophysiological characteristics of SDT rats.

### MATERIALS AND METHODS

### Establishment of the SDT Rat

In 1988, five male rats with polyuria and glucosuria were recognized among 305 rats from an outbred colony of the Crj : CD(SD) strain (Charles River Japan, Inc., Kanagawa, Japan) of Sprague-Dawley rats. Polyphagia and polydipsia were also noted in the affected rats, which were kept in the Research Laboratories of Torii Pharmaceutical Co., Ltd., Chiba, Japan. After 20th generation of sister – brother mating, the diabetic strain was established in 1997, and named the Spontaneously Diabetic Torii (SDT) rat. SDT rats were maintained in a specific pathogen-free (SPF) facility in our laboratory, especially with regard to the following microorganisms: Sendai virus, Sialodacryoadenitis virus, the pneumonia virus of mice, mouse encepalomyelitis virus, Kilham rat virus, H-1 virus, the minute virus of mice, Hantavirus, mouse adenovirus, *Mycoplasma pulmonis, Bacillus piliformis, CAR bacillus, Bordetella bronchiseptica, Corynebacterium kutscheri, Pasteurella pneumotropica, Pseudomonas aeruginosa, Salmonella spp, Salmonella typhimurium, Streptococcus pneumoniae, Dermatophytes, Giardia muris, Spironucleus muris* and *Syphacia spp.*

### Animals

The SDT rats were raised at the Research Laboratories of Torii Pharmaceutical Co., Ltd. Crj : CD(SD) rats were purchased from Charles River Japan, Inc., and were used as reference animals in this study. All rats were maintained under SPF conditions at $23 \pm 2°C$, $55 \pm 10\%$ relative humidity, a 12-h light-dark cycle, and provided with a commercial pellet (CE-2, Clea Japan Ltd., Tokyo, Japan) and water *ad libitum* from an automatic sterilized water supply system. All animals were handled in accordance with the recommendations of the Animal Welfare Committee of the Research Laboratories of Torii Pharmaceutical Co., Ltd. The body weight and plasma glucose levels were measured once every 2 weeks from 6 to 20 weeks of age, and thereafter, at 5-week intervals. The urine volume was also determined.

### Incidence of Diabetes and Survival Rate

Both in male ($n = 42$) and female ($n = 39$) SDT rats, the urinary glucose and keto-body contents were determined using Multstix® (Bayer–Sankyo Co., Tokyo, Japan), every week between 5 and 65 weeks of age, to determine the cumulative incidence of DM and the survival rate. The rats were diagnosed as being diabetic if the urinary glucose was 3+ or higher.

### Oral Glucose Tolerance Test (OGTT)

The OGTT was performed after 16 hr of fasting in male SDT rats and male Crj : CD(SD) rats at 12 weeks ($n = 6$) and 16 weeks ($n = 6$) of age, with 2 g/kg of glucose. Blood samples were collected from the jugular vein before the administration of glucose, and 30, 60 and 120 min after the oral glucose administration. Plasma glucose levels were measured by the glucose oxidase method using Glucose-Test Wako® (Wako Pure Chemical Co., Osaka, Japan).

### Biochemical Analysis

Male SDT rats and male Crj : CD(SD) rats were placed in metabolic cages under a non-fasting condition, and 18-hr urine samples were collected. Blood samples were drawn from the abdominal aorta of the animals under ether anesthesia into tubes with the anticoagulant dipotassium EDTA for hematological tests, and heparin for plasma biochemical analysis. The heparinized blood samples were centrifuged at 3,500 rpm for 15 min, and the supernatant plasma frozen at −80°C until analysis. Hemoglobin A1c (HbA1c) level in the blood was measured using a HbA1c analyzer (DCA2000+, Bayer–Sankyo Co.). Urinary protein concentration was determined by the pyrogallol-red method (Micro TP-Test Wako®, Wako Pure Chemical Co.). Plasma glucose (GLU), triglyceride (TG), total cholesterol (T-CHO) and urea nitrogen (UN) levels were measured with an automatic analyzer (Hitachi-7150, Hitachi Co., Tokyo, Japan). Plasma level of immunoreactive insulin was measured by enzyme-linked immunosorbent assay (ELISA) using a rat insulin measurement kit (Morinaga Institute of Biological Science, Yokohama, Japan).

### Histopathological Examinations

The pancreas in 10-, 12-, 14-, 16-, 18-, 20-, 25-, 30-, 35-, 40-, 70- and 77-week-old rats were weighed and fixed in 10% neutral buffered formalin. In addition, their eyes were fixed in a mixture of glutaraldehyde and formalin. The fixed specimens were embedded in paraffin, sectioned at 4 μm, and stained with hematoxylin and eosin (H&E) for histopathological examination.

### Statistical Analysis

The data are given as mean ± SD. The differences between the SDT and age- and sex-matched Crj : CD(SD) rats were compared by the Student's $t$-test. Probability values of $P < 0.05$ were considered as denoting significance.

### RESULTS

### Body Weight and Non-fasting Plasma Glucose Levels

The changes in body weight of male and female SDT and Crj : CD(SD) rats are shown in Figure 1. The average body weight of male SDT rats was slightly lower than that of Crj : CD (SD) rats until 18 weeks of age, and then gradually decreased as the incidence of diabetes increased. A significant decrease in body weight was observed from 20 weeks of age (Fig. 1A). On the other hand, the average body weight of female SDT rats from 6 to 40 weeks of age increased gradually throughout the experimental period, and the changes in body weight in female SDT rats were similar to those in female Crj : CD(SD) rats (Fig. 1B). The changes in non-fasting plasma glucose levels in male and female SDT and Crj : CD(SD) rats are also shown in

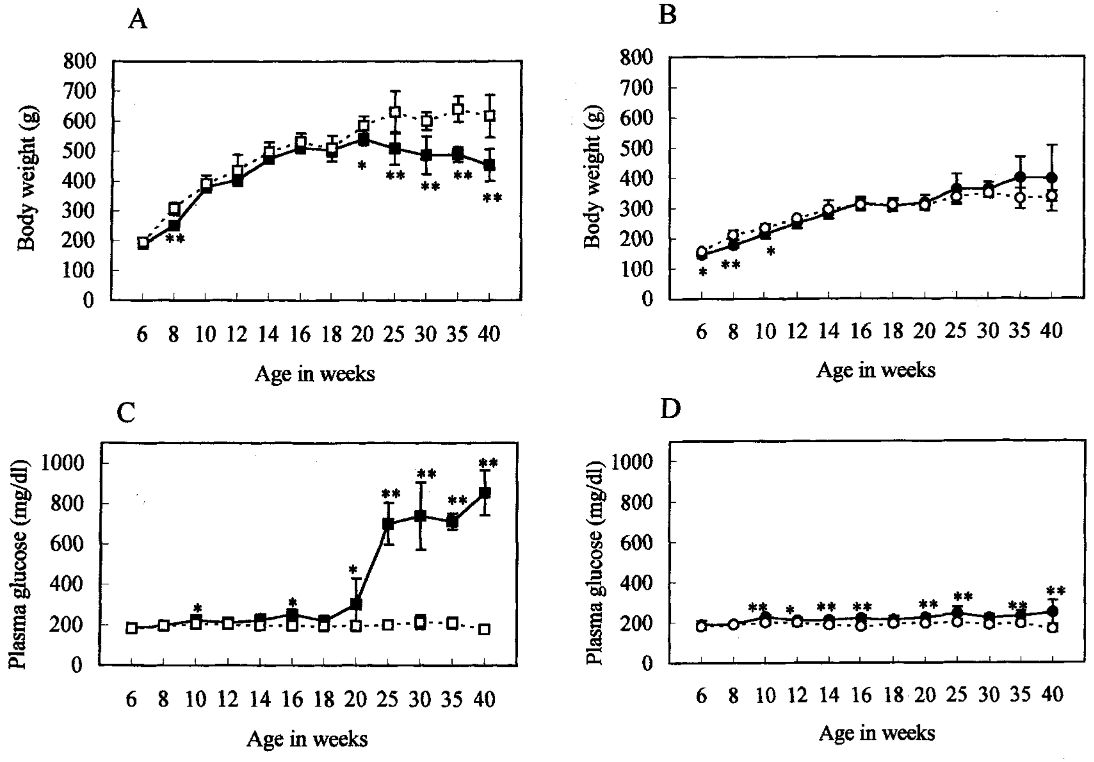

FIGURE 1 Changes in body weight (A: male, B: female) and plasma glucose levels (C: male, D: female) in SDT rats and Crj:CD(SD) rats, from 6 to 40-week-old. ■, male SDT; ●, female SDT; □, male Crj:CD(SD); ○, female Crj:CD(SD). Each value represents mean ± SD. of 5–8 rats for each experimental time point. *$P<0.05$, **$P<0.01$ *vs.* Crj:CD(SD) rats.

Figure 1. The non-fasting plasma glucose levels in male SDT rats reached 701 ± 103 mg/dl by 25 weeks of age ($P < 0.01$) and the values gradually increased with advancing ages, whereas those in Crj : CD(SD) rats were maintained at a steady level (Fig. 1C). Plasma glucose levels in female SDT rats were slightly but significantly higher than those in female Crj : CD(SD) rats after 10 weeks of age (Fig. 1D).

### Incidence of Diabetes

The cumulative incidences of diabetes in male and female SDT rats are shown in Figure 2. Glucosuria appeared at 20-week-old in male SDT rats (15/42). The time of onset of glucosuria was coincident with that of plasma glucose elevation. The cumulative incidence of diabetes increased sharply to 52.4% in 25-week-old rats (22/42) and 100% in 40-week-old rats (42/42). Glucosuria had not yet appeared even by 40-week-old in female SDT rats. It was first observed at 45-week-old, and thereafter, the incidence of diabetes gradually increased. However, the maximal incidence of 33.3% noted in female rats at 65 weeks of age was markedly lower than that noted in male SDT rats. In both sexes of SDT rats after the onset of diabetes, the symptoms of polyuria, polydipsia and polyphagia were also observed. However, no ketonuria was noted up to 65 weeks of age in male and female SDT rats.

### Biochemical Parameters and Organ Weight

The changes in biochemical parameters and organ weight in male SDT rats and male Crj : CD(SD) rats are shown in Table I. The pancreas weights of male SDT rats were significantly lower ($P < 0.01$) than those in Crj : CD(SD) rats at 25 weeks of age. Plasma levels of insulin in male SDT rats gradually decreased from 10-week-old. The plasma insulin levels of male SDT rats were significantly lower ($P < 0.01$) than those in 25-week-old Crj : CD(SD) rats and marked hyperglycemia (701 ± 103 mg/dl) in the non-fasting state was associated with this hypoinsulinemia. Hypertriglyceridemia was also noted in male 35-week-old SDT rats. Plasma levels of triglyceride, in male SDT rats were 2.7 times higher ($P < 0.01$) than those in Crj : CD(SD)

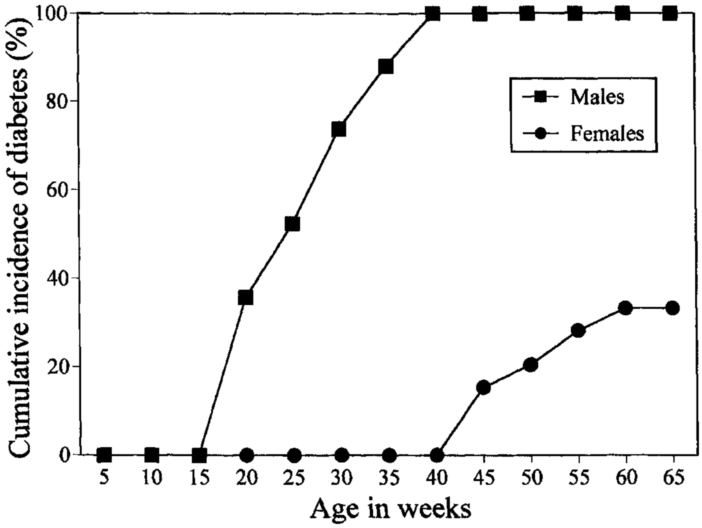

FIGURE 2 Cumulative incidences of diabetes in SDT rats. ■, Male ($n = 42$); ●, Female ($n = 39$).

TABLE I Biochemical parameters and pancreas weight in male SDT and Crj:CD(SD) rats

| | SDT | | | Crj:CD(SD) | | |
|---|---|---|---|---|---|---|
| Age in weeks (No. of animals) | 10 (7) | 25 (7)† | 35 (5)† | 10 (6) | 25 (6) | 35 (6) |
| Pancreas weight (g) | 1.2±0.1 | 1.1±0.1** | 1.1±0.1* | 1.3±0.1 | 1.4±0.2 | 1.5±0.3 |
| Plasma insulin (ng/ml) | 2.8±1.2 | 1.2±0.8** | 0.4±0.2** | 3.4±1.0 | 3.1±1.2 | 3.2±1.3 |
| Plasma triglyceride (mg/dl) | 64±8** | 146±71 | 214±54** | 90±19 | 95±35 | 78±20 |
| Plasma total cholesterol (mg/dl) | 57±5 | 72±15 | 87±24 | 56±7 | 62±11 | 61±6 |
| Plasma urea nitrogen (mg/dl) | 18.8±3.0 | 26.7±4.3 | 30.4±3.0** | 23.6±0.5 | 22.3±3.6 | 21.1±4.2 |
| UPE (mg/18 hr) | 17.6±1.5 | 13.9±7.4 | 62.1±24.5** | 20.2±4.9 | 16.7±6.6 | 16.0±4.0 |
| HbA1c (%) | – | – | 9.2±1.1** | – | – | 2.6±0.1 |

UPE: Urinary protein excretion. Each value represents the mean ±SD.
*,** Significant difference from the age-matched Crj:CD(SD) rat group values at $P<0.05$, $P<0.01$, respectively.
†All rats in this group were observed with diabetes.

rats in this age group. Furthermore, the plasma urea nitrogen levels, urinary protein excretion and HbAlc levels in male SDT rats were 1.4, 3.9 and 3.5 times higher respectively ($P<0.01$) than those in Crj:CD(SD) rats at 35 weeks of age. The plasma total cholesterol levels remained unchanged in male SDT rats at 10, 25 and 35 weeks of age.

### Survival Rate

The survival rates of male and female SDT rats up to 65-week-old were 92.9% (39/42) and 97.4% (38/39), respectively. The first death among male and female SDT rats was observed at 40-week-old and 45-week-old, respectively. In the mortality of diabetic males (3/42), enlarged kidneys were macroscopically observed, and dilation of uriniferous tubules, accumulation of glycogen in the epithelial cells of the uriniferous tubules, interstital inflammatory cell infilitration, and hyaline casts were histologically observed. Since these changes were not observed in the mortality of females (1/39) without DM.

### OGTT

The results of the OGTT in 12 and 16-week-old male SDT rats and male Crj:CD(SD) rats are shown in Table II. Slight glucose intolerance was first noted in SDT male rats when 12-week-old. When 16-week-old, the fasting plasma glucose levels in male SDT rats were similar to those in Crj:CD(SD) rats. However, the plasma glucose levels at 30, 60 and 120 min after glucose administration in male SDT rats (16w) were significantly ($P<0.01$) increased compared with those in Crj:CD(SD) rats, indicating marked glucose intolerance induced in male 16-week-old SDT rats.

TABLE II Oral glucose tolerance test in male SDT and Crj:CD(SD) rats at 12 and 16 weeks of age

| | | | Plasma glucose (mg/dl) | | | |
|---|---|---|---|---|---|---|
| Age in weeks | Strain | No. of animals | 0 min | 30 min | 60 min | 120 min |
| 12 | Crj:CD(SD) | 6 | 130±28 | 205±24 | 184±21 | 128±18 |
| | SDT | 6 | 107±12 | 252±39* | 219±43 | 150±23 |
| 16 | Crj:CD(SD) | 6 | 123±14 | 178±26 | 202±8 | 150±17 |
| | SDT | 6 | 116±14 | 266±24** | 317±51** | 223±63* |

Each value represents the mean ± SD.
*,** Significant difference from the age-matched Crj:CD(SD) rat group values at $P<0.05$, $P<0.01$, respectively.

### Histopathological Findings

The histopathological features in the pancreas of male SDT rats were as follows: in 10–20-week-old, slight changes such as hemorrhage, hemosiderin deposition, inflammatory cell infiltration and fibrosis in and around the islets; in 25-week-old, hemosiderin deposition, cellular infiltration with lymphocytes and macrophages, and fibrous tissue proliferation in and around the pancreatic islets, dividing the islets into small lobules (Fig. 3). By 30–40-weeks, hemosiderin deposition and fibrosis were observed in and around the islets. In addition, hyperplasia of the small pancreatic ducts in and around the islets was observed in 25–40-week-old animals. The size and number of islets were further decreased. After 40-weeks, the islets were much smaller, and partially replaced by connective tissue. Both the number and size of the islets were further decreased and eventually absent. Macroscopic opacity of the lens was observed at 40 weeks of age or older in diabetic male SDT rats. Histopathologically, swelling, vacuolation and disintegration of the lens fibers, and formation of Morgani's globules were observed. In the retina, tractional retinal detachment with fibrous proliferation in the direction of the vitreous cavity, and thickening and distortion of the retina were observed in diabetic male SDT rats at 70 weeks of age (Figs. 4,5). However, marked retinal hemorrhage was not observed in most case. In 77-week-old male SDT rats, massive hemorrhaging in the anterior chamber of the eye was observed in

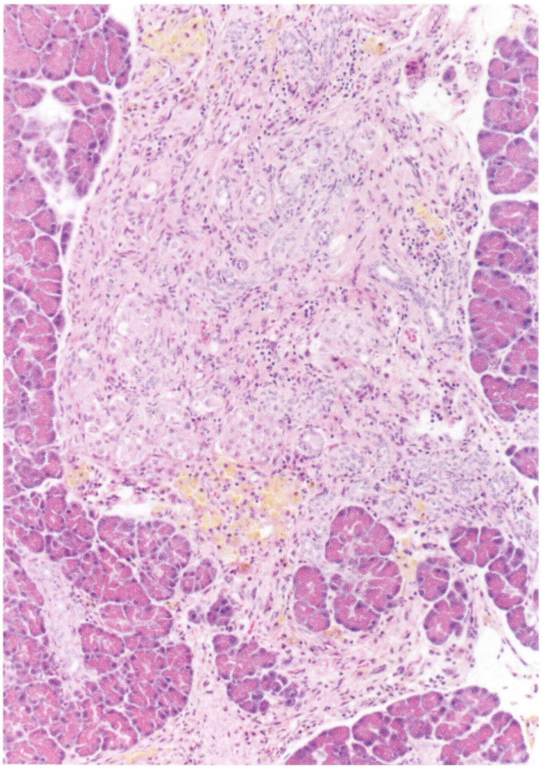

FIGURE 3 Histopathological changes in the pancreas of a male SDT rat at 25-week-old. Infiltration and fibrosis in and around the pancreatic islets are seen (H&E stain, × 120). (See Color Plate I).

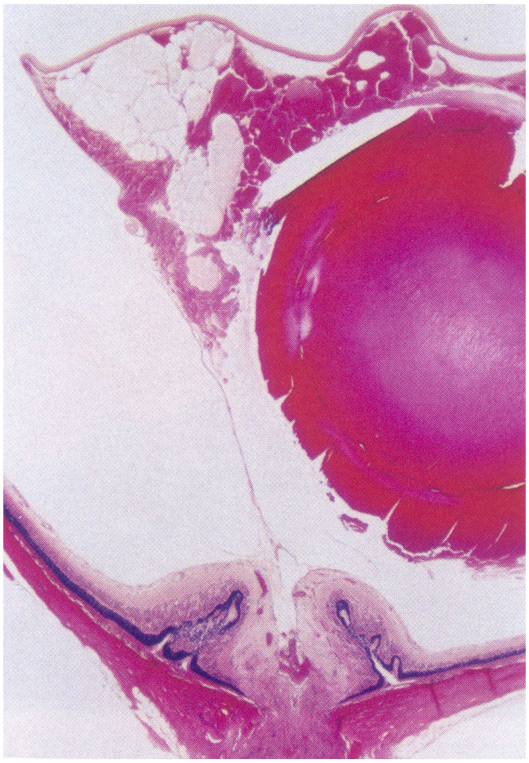

FIGURE 4 Histopathological changes in the lens and retina of a male SDT rat at 70-week-old. Swelling, vacuolation and disintegration of the lens fibers, and fibrous proliferation in the direction of the vitreous cavity are observed (H&E stain, × 30). (See Color Plate II).

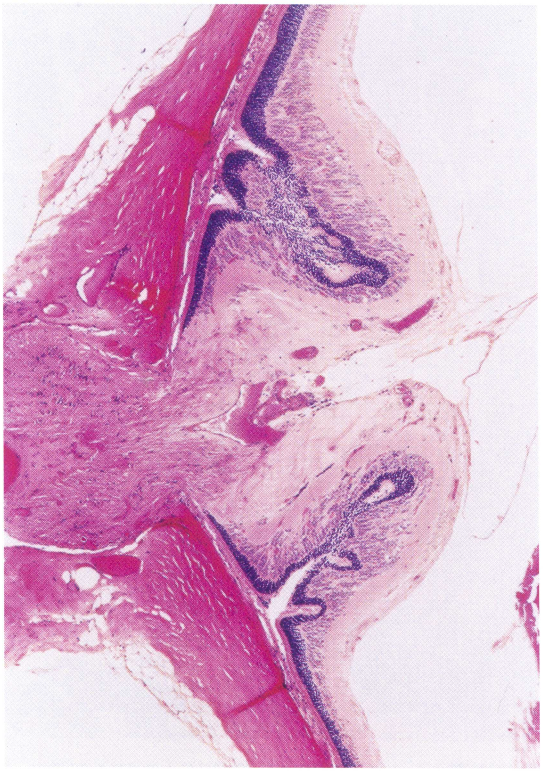

FIGURE 5 Histopathological changes in the retina of a male SDT rat at 70-week-old. Tractional retinal detachment, thickening and distortion are observed (H&E stain, ×73). (See Color Plate III).

some cases (Fig. 6). This massive hemorrhage was associated with fibrous proliferation around the iris (Fig. 7A, B).

### DISCUSSION

In the present study, glucosuria, polyuria and polydipsia were observed in male SDT rats after 20 weeks of age and the nonfasting plasma levels of insulin, glucose and triglyceride began to change after 25 weeks. Clinical diabetes was first confirmed in male SDT rats of this age. The SDT strain of rats was separated from outbred Crj:CD(SD) rats, and this strain was used as a reference to the SDT strain. The body weight of male SDT rats was significantly lower than those of sex- and age-matched Crj:CD(SD) rats. These data suggest that our SDT rats should be classified as non-obese diabetic animals. The non-fasting plasma glucose level in male SDT rats reached approximately 700 mg/dl when 35-weeks. The non-fasting plasma insulin level (0.4 ng/ml) was significantly lower than that in Crj:CD(SD) rats of the same age. Furthermore, hypertriglyceridemia was associated with the hyperglycemia. Based on these data, it is suggested that SDT rats belong to the class of diabetic animal models with severe hyperglycemia and hypoinsulinemia.

It was reported that insulin treatment was required for the survival of spontaneously Type 1 diabetic rats such as BB,[5] LETL[7] and KDP[8] rats. However, SDT rats survived for a long time even without insulin treatment. Ketonuria was noted in BB rats[15] but not in SDT rats. It was reported that the appearance of persistent ketonuria associated with hyperglycemia or severe glucosuria in a diabetic patient points to an unacceptably severe level of metabolic disturbance and indicates an urgent need for corrective action.[16] From these data, it is suggested that glucose and lipid metabolism might be differently disturbed in SDT and BB rats.[5,17] In SDT rats, the cumulative incidence of diabetes was shown to be 100% in male rats by 40-week-old and only 33.3% in female rats even by 65-week-old. Sex difference was similarly observed in Wistar Fatty and OLETF rats.[13,14]

To clarify the type of DM in the SDT rat, morphological examination of the pancreas was carried out. Histopathological examination revealed hemorrhage in and around the islets by about 10-week-old followed by inflammatory cell infiltration and fibrosis around the islets, resulting in a significantly decreased number and size of the islets in 40-week-old animals. Although these time-dependent histopathological changes, in particular, the morphological pattern of fibrosis around the islets, resembled those observed in WBN/Kob rats,[18–20] inflammation of the exocrine pancreas was considered to be less prominent than that in WBN/Kob rats.[18–20] The Type 1 diabetic BB rat was characterized by prominent insulitis and lymphopenia, namely deficiency of T lymphocytes,[21]

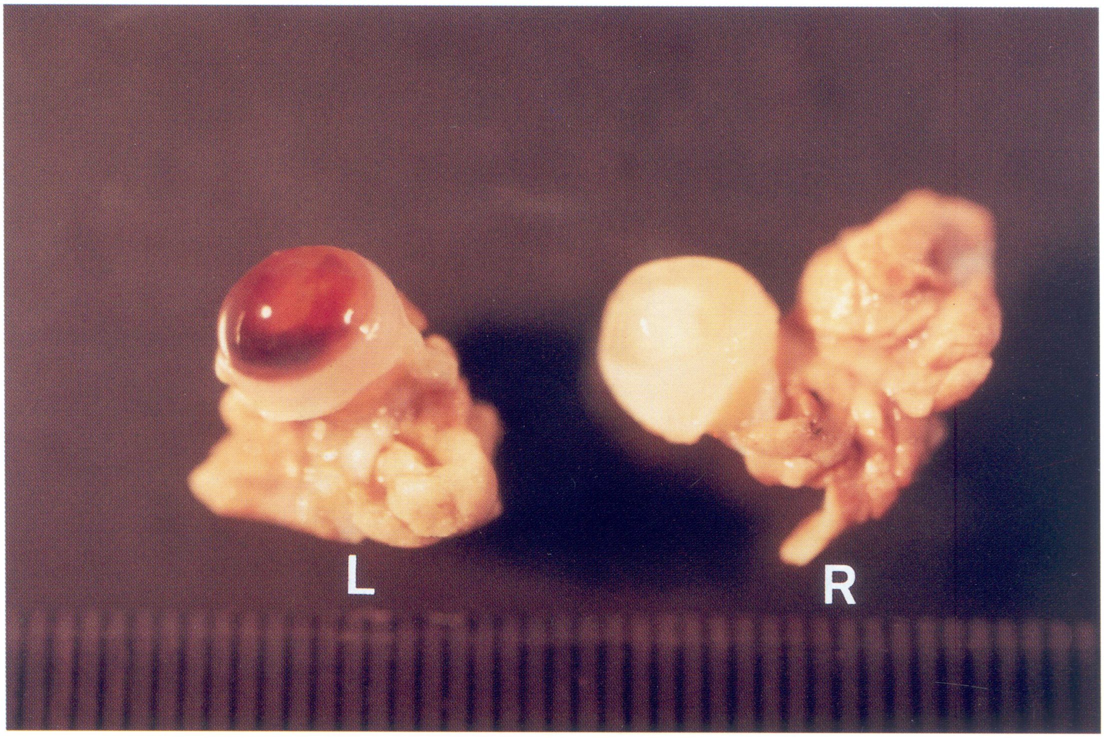

FIGURE 6 Massive hemorrhage in the anterior chamber observed in a male SDT rat at 77-week-old macroscopically. The massive hemorrhage was observed in the left eye (L) but not in the right eye (R). (See Color Plate IV).

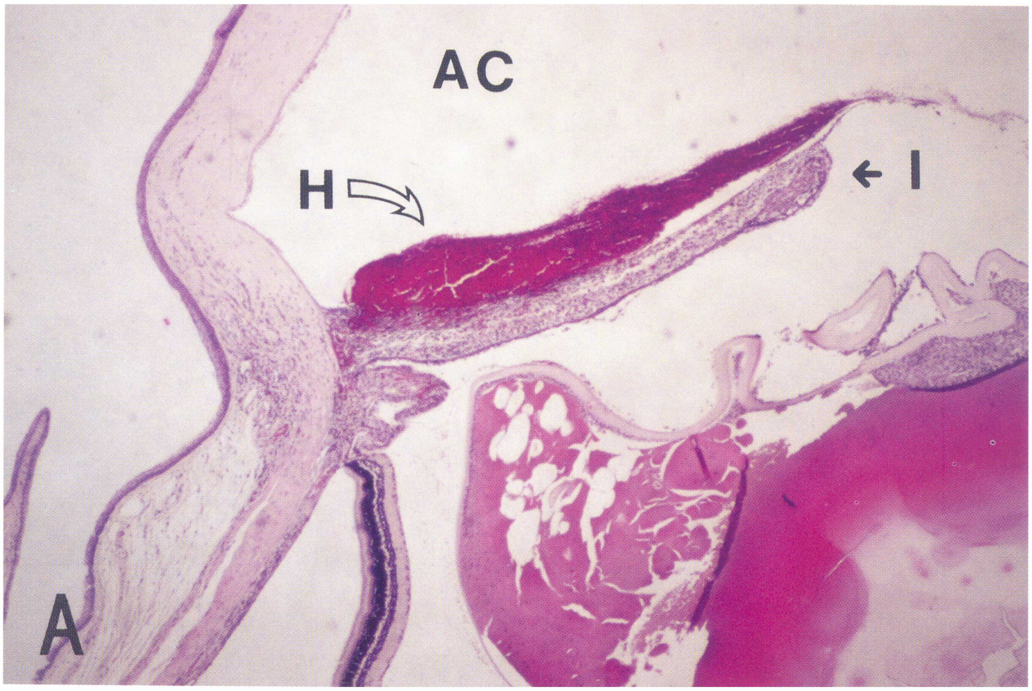

FIGURE 7 Histopathological changes in the iris of a male SDT rat in Figure 6. A: massive hemorrhage (H) associated with fibrous proliferation over the iris (I). B: higher magnification of the portion of massive hemorrhage in Figure 7A. AC: anterior chamber. A (H&E stain, × 100); B (H&E stain, × 475). (See Color Plate V).

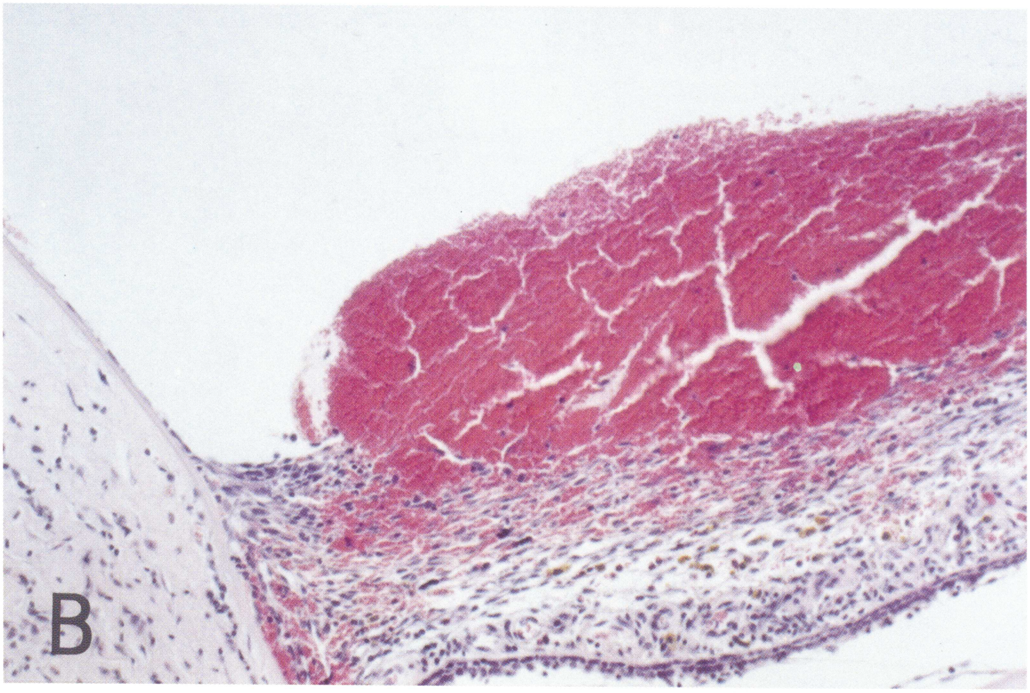

FIGURE 7 (Continued). (See Color Plate V).

however, such insulitis and lymphopenia were not observed in the SDT rat. The histological findings and clinical features suggest that the SDT rat should be classified as a Type 2 DM model. Recently, new diabetic mouse models such as Akita mice [22] and TSOD mice [23] have been reported. However, new spontaneously diabetic models have not been reported in rats. Therefore, it is interest that the SDT rat, established by us, has a disease resembling human non-obese severe Type 2 diabetes with insulin hyposecretion.

Various complications of diabetes have already been reported in spontaneously diabetic animal models. As for ocular lesions, cataract was observed in WBN/Kob rats at 15–24 months. [24] Cataract is one of the ocular complications observed in diabetic patients. [25] In the present study, in diabetic male SDT rats, macroscopic opacity of the lens with histopathological changes such as swelling, vacuolation and disintegration of the lens fibers, and formation of Morgani's globules were observed. These abnormalities were also observed in the aged WBN/Kob rats, [26] however, SDT rats showed these changes at an earlier age. Among the various ocular complications in diabetes, the most serious is retinopathy. Since it produces more blindness that any other ocular involvement of diabetes. According to the classification of Davis *et al.*, human diabetic retinopathy is mainly classified into nonproliferative or proliferative retinopathy. [27] It was reported that morphological changes like human diabetic retinopathy were induced in some animal models. For example, the spontaneously diabetic BB rats, [28] galactosemic dogs, [29] storeptozotocin-diabetic rats [30] and oxygen-induced retinopathic mice [31] are well known. However, there are no other reports of lesions resembling human proliferative retinopathy than oxygen-induced retinopathic models, so useful spontaneously proliferative retinopathic models must be developed. Morphologically, diabetic male

SDT rats showed tractional retinal detachment with fibrous proliferation, thickening and distortion of the retina by 70 weeks of age. These changes resemble those seen in human proliferative retinopathy. Since these changes were not observed in age-matched Crj : CD(SD) rats or age-matched female SDT rats without DM, it is suggested that the ocular lesions in SDT rats might be caused by diabetic condition especially chronic hyperglycemic stress. However, no significant retinal hemorrhage was observed in most cases. The fibrous proliferation did not show significant neovascular formation. On the other hand, massive hemorrhage in the anterior chamber of the eye was observed in diabetic males. The massive hemorrhage associated with fibrous proliferation around the iris was observed histopathologically. These findings correspond to the hemorrhage that resulted from iris neovascularization, which is frequently complicated with severe untreated proliferative diabetic retinopathy. Although a low incidence of retinal hemorrhage and poor development of neovascular formation in the proliferative tissue over the retina did not match the typical human proliferative diabetic retinopathy, other ocular changes observed in SDT rats, *i.e.*, cataract, proliferative changes of the vitreous and the retina, and massive hemorrhage in the anterior chamber, resemble those seen in human proliferative diabetic retinopathy. However, in this study, only conventional histopathologic standing with hematoxylin-eosin was done to evaluate the ocular changes. To obtain a better understanding of retinopathy in the SDT rat, analysis of the retinal vasculature using a trypsin digest technique and fluorescein-dextran angiography are required.

Development of a new diabetic model which can exhibit the complication of diabetic retinopathy resembling that in humans would be rather useful to clarify the cause of human diabetic retinopathy and to develop treatment methods. Our research has also paved the way for new approaches in human diabetic retinopathy research. If factors predictive of potential diabetic retinopathy can be found in SDT rats, these findings can be utilized to advantage in humans. We believe that the SDT rat is a highly useful model for strengthening our understanding of the disease process of diabetic complications in humans.

As described above, we have established this diabetic strain and clarify its diabetic characteristics. However, our colony of SDT rats is still too small to make the rats available to diabetes researchers. Our much effort will be required to make the colony big enough.

### *Acknowledgments*

We are grateful to Dr. Yoshitaka Ino, Dr. Toru Ando, Kazuyoshi Watanabe, Shoshi Suzuki, Masateru Kurumi and Taro Ushijima for their invaluable advice during the course of this study and Yuji Yamazaki, Takuya Abe and Tomohisa Seo for excellent technical assistance.

### *References*

[1] King, H. and Zimmet, P. (1988). Trend in the prevalence and incidence of diabetes: Non-insulin-dependent diabetes mellitus. *Wld. Hlth. Statist. Quart.*, **41**, 190–196.

[2] King, H. and Rewers, M. (1991). WHO Ad Hoc Diabetes Reporting Group. Diabetes in adult is now a Third World problem. *WHO Bulletin OMS*, **69**, 643–648.

[3] King, H. and Rewers, M. (1993). WHO Ad Hoc Diabetes Reporting Group. Global estimates for prevalence of diabetes mellitus and impaired glucose tolerance in adults. *Diabetes Care*, **16**, 157–177.

[4] Amos, A. F., McCarty, D. J. and Zimmit, P. (1997). The rising global of diabetes and its complications: estimates and projections to the year 2010. *Diabetes Med.*, **14**, S7–S85.

[5] Nakhooda, A. F., Like, A. A., Chapple, C. I., Murray, F. T. and Marliss, E. B. (1977). The spontaneously diabetic Wistar rat. Metabolic and morphologic studies. *Diabetes*, **26**, 100–112.

[6] Makino, S., Kunimoto, K., Muraoka, Y., Mizushima, Y., Katagiri, K. and Tochino, Y. (1980). Breeding of a non-obese, diabetic strain of mice. *Exp Anim.*, **29**, 1–13.

[7] Kawano, K., Hirashima, T., Mori, S., Saitoh, Y., Kurosumi, M. and Natori, T. (1991). New inbred strain of Long-Evans Tokushima Lean Rats with IDDM without lymphopenia. *Diabetes*, **40**, 1375–1381.

[8] Yokoi, N., Kanazawa, M., Kitada, K., Tanaka, A., Kanazawa, Y., Suda, S., Ito, H., Serikawa, T. and

Komeda, K. (1997). A non-MHC locus essential for autoimmune type I diabetes in the Komeda diabetes-prone rat. *J. Clin. Invest.*, **100**, 2015–2021.

[9] Colman, D. L. and Hummel, K. P. (1973). The influence of genetic background on the expression of the obese (ob) gene in the mouse. *Diabetologia*, **9**, 287–293.

[10] Dulin, W. E. and Wyse, B. M. (1970). Diabetes in the KK mouse. *Diabetologia*, **6**, 317–323.

[11] Colman, D. L. and Hummel, K. P. (1974). Hyperinsulinemia in pre-weaning diabetes (db) mice. *Diabetologia*, **10**, 607–610.

[12] Goto, Y., Suzuki, K., Sasaki, M., Ono, T. and Abe, S. (1988). GK rats as a model of nonobese, noninsulin-dependent diabetes: selective breeding over 35 generations. In: *Frontiers in Diabetes Research: Lessons from Animal Diabetes II*, edited by Shafrir, E. and Renold, A. E., pp. 301–303. London, John Libbey.

[13] Ikeda, H., Shino, A., Matsuo, T., Iwatsuka, H. and Suzuoki, Z. (1981). A new genetically obese-hyperglycemic rat (Wistar Fatty). *Diabetes*, **30**, 1045–1050.

[14] Kawano, K., Hirashima, T., Mori, S., Saitoh, Y., Kurosumi, M. and Natori, T. (1992). Spontaneous long-term hyperglycemic rat with diabetic complications. Otsuka Long-Evans Tokushima Fatty (OLETF) Strain. *Diabetes*, **41**, 1422–1428.

[15] Chappel, C. I. and Chappel, W. R. (1983). The discovery and development of the BB rat colony: an animal model of spontaneous diabetes mellitus. *Metabolism*, **32** (Suppl. 1), 8–10.

[16] Kitabchi, A. and Rumbak, M. (1989). The management of diabetic emergencies. *Hosp. Prac.*, **24**, 129–133.

[17] Nakhooda, A. F., Like, A. A., Chapple, C. I., Wei, C. N. and Marliss, E. B. (1978). The spontaneously diabetic Wistar rat (the "BB" rat). Studies prior to and during development of the overt syndrome. *Diabetologia*, **14**, 199–207.

[18] Tsuchitani, M., Saegusa, T., Narama, I., Nishikawa, T. and Gonda, T. (1985). A new diabetic strain of rat (WBN/Kob). *Lab. Anim.*, **19**, 200–207.

[19] Nakama, K., Shichinohe, K., Kobayashi, K., Naito, K., Uchida, O., Yasuhara, K. and Tobe, M. (1985). Spontaneous diabetes-like syndrome in WBN/Kob rats. *Acta. Diabetol. Lat.*, **22**, 335–342.

[20] Mori, Y., Yokoyama, J., Nishimura, M., Kurata, H., Miura, J. and Ikeda, Y. (1990). Diabetic strain (WBN/Kob) of rat characterized by endocrine-exocrine pancreatic impairment due to distinct fibrosis. *Pancreas*, **5**, 452–459.

[21] Greiner, D. L., Handler, E. S., Nakano, K., Mordes, J. P. and Rossini, A. A. (1986). Absence of the RT-6 T cell subset in diabetes-prone BB/W rats. *J. Immunol.*, **136**, 148–151.

[22] Yoshioka, M., Kayo, T., Ikeda, T. and Koizumi, A. (1997). A novel locus, Mody4, distal to D7Mit189 on chromosome 7 determines early-onset NIDDM in nonobese C57BL/6 (Akita) mutant mice. *Diabetes*, **46**, 887–894.

[23] Suzuki, W., Iizuka, S., Tabuchi, M., Funo, S., Yanagisawa, T., Kimura, M., Sato, T., Endo, T. and Kawamura, H. (1999). A new mouse model of spontaneous Diabetes derived from ddY strain. *Exp. Anim.*, **48**, 181–189.

[24] Mori, Y., Yokoyama, J., Nishimura, M., Oka, H., Mochino, S. and Ikeda, Y. (1992). Development of diabetic complications in a new diabetic strain of rat (WBN/Kob). *Pancreas*, **7**, 569–577.

[25] Bursell, S. E., Kalalekas, D. P. and Craig, M. S. (1989). The effect of acute changes in blood glucose on lenses in diabetic and non-diabetic subjects using quasi-elastic light scattering spectroscopy. *Current Eye Res.*, **8**, 821–834.

[26] Miyamura, N. and Amemiya, T. (1998). Lens and retinal changes in the WBN/Kob rat (spontaneously diabetic strain). Electron-microscopic study. *Opthalmic Res.*, **30**, 221–232.

[27] Davis, M. D., Myers, F. L., Bresnick, G. H. and Venecia, G. D. (1977). Natural evolution. In: *Current Diagnosis and Management of Chorioretinal Diseases*, pp. 179–184, L'Esperance FA, (Ed.), Saint Louis, The C. V. Mosby Company.

[28] Sima, A. A. F., Chakrabatri, S., Garcia-Salinas, R. and Basu, P. K. (1985). The BB-rat – an authentic model of human diabetic retinopathy. *Curr. Eye. Res.*, **4**, 1087–1092.

[29] Engerman, R. L. and Kern, T. S. (1984). Experimental galactosemia produces diabetic-like retinopathy. *Diabetes*, **33**, 97–100.

[30] Tilton, R. G., LaRose, L. S., Kilo, C. and Williamson, J. R. (1986). Absence of degenerative changes in retinal and uveal capillary pericytes in diabetic rats. *Invest. Ophthalmol. Vis. Sci.*, **27**, 716–721.

[31] Smith, L. E. H., Wesolowski, E., McLellan, A., Kostyk, S. K., D'Amato, R., Sullivan, R. and D'Amore, P. A. (1994). Oxygen-induced retinopathy in the mouse. *Invest. Ophthalmol. Vis. Sci.*, **35**, 101–111.

